# iTRAQ-Based Quantitative Proteomic Analysis of *Spirulina platensis* in Response to Low Temperature Stress

**DOI:** 10.1371/journal.pone.0166876

**Published:** 2016-11-30

**Authors:** Qingye Li, Rong Chang, Yijun Sun, Bosheng Li

**Affiliations:** 1 College of Biological Sciences and Technology, Beijing Forestry University, Haidian District, Beijing, China; 2 College of Nature Conservation, Beijing Forestry University, Beijing, China; 3 Institute of *Spirulina* Research, Beijing Forestry University, Beijing, China; Universidade Federal de Vicosa, BRAZIL

## Abstract

Low temperature (LT) is one of the most important abiotic stresses that can significantly reduce crop yield. To gain insight into how *Spirulina* responds to LT stress, comprehensive physiological and proteomic analyses were conducted in this study. Significant decreases in growth and pigment levels as well as excessive accumulation of compatible osmolytes were observed in response to LT stress. An isobaric tag for relative and absolute quantitation (iTRAQ)-based quantitative proteomics approach was used to identify changes in protein abundance in *Spirulina* under LT. A total of 3,782 proteins were identified, of which 1,062 showed differential expression. Bioinformatics analysis indicated that differentially expressed proteins that were enriched in photosynthesis, carbohydrate metabolism, amino acid biosynthesis, and translation are important for the maintenance of cellular homeostasis and metabolic balance in *Spirulina* when subjected to LT stress. The up-regulation of proteins involved in gluconeogenesis, starch and sucrose metabolism, and amino acid biosynthesis served as coping mechanisms of *Spirulina* in response to LT stress. Moreover, the down-regulated expression of proteins involved in glycolysis, TCA cycle, pentose phosphate pathway, photosynthesis, and translation were associated with reduced energy consumption. The findings of the present study allow a better understanding of the response of *Spirulina* to LT stress and may facilitate in the elucidation of mechanisms underlying LT tolerance.

## Introduction

*Spirulina platensis* is a filamentous, multicellular, non-heterocyst-forming spiral algae. It contains high levels of protein, low amounts of fat and cholesterol, as well as various essential amino acids, micronutrients, and biological active substances such as phycocyanin, β-carotene, and γ-linolenic acid that provide health benefits to the human body [1–3]. Previous studies have shown that it increases immunity, as well as possesses anti-tumor, anti-radiation, antioxidant, and antihypertensive effects [4–7]. *Spirulina* is widely used in functional food, cosmetics, feed, and pharmaceuticals [8]. *Spirulina* has a strong ability to adapt to adverse conditions such as highly alkaline and saline environments, as well as resist extreme temperatures and radiation. Based on these features, *Spirulina* may be a good model organism for investigating adaptations to changes in the environment.

Variations in temperature are a common stress for various living organisms, including cyanobacteria. In the northern region of China, *Spirulina* production is mainly performed outdoors. In spring and autumn, outdoor temperature undergoes extensive fluctuations, ranging from 15°C in the early morning to 30°C at midday. At 15°C, the growth rate of *Spirulina* markedly decreases and then slowly reverts back to its normal rate, thus significantly affecting its productivity [9–11]. Response mechanisms that protect against potentially harmful effects of low temperature have been extensively studied in *Spirulina*; however, most of these studies are focused on physiological responses and specific genes [12–15]. For example, homeoviscous adaptation, which involves the induction of polyunsaturated fatty acids synthesis at Low temperature (LT), plays a significant role in cold adaptation in cyanobacteria, including *Spirulina* [16,17]. Lee-Feng et al. [18] demonstrated that enhanced antioxidant enzyme activities at LT (15°C) are an efficient regulatory strategic response to LT-induced photoinhibition. In addition, a previous study also indicated that the adaptation of *Arthrospira platensis* to cold stress involves a down-regulation of photosynthetic activity and increased resistance to photoinhibition [19].

In recent years, proteomics analysis has become an effective approach in identifying a wide range of differentially expressed proteins and in investigating the molecular mechanisms underlying the response of various organisms to different environmental stresses. Several research studies involving the proteomics analyses of plants (wheat, barley, rice, and *Arabidopsis*) have indicated that plants utilize common adaptive responses to LT stress, which include the down-regulation of photosynthesis-related proteins and up-regulation of proteins involved in carbohydrate metabolism, ROS scavenging, cell wall remodeling, cytoskeletal rearrangements, and detoxification [20–23]. Hongsthong et al. [24,25] examined the changes in the proteomics profile of *Spirulina* under LT (22°C) at the sub-cell level, which revealed that proteins involved in two-component response systems, DNA repair, molecular chaperones, secretion systems, and nitrogen assimilation play important roles in the response of *Spirulina* in cold stress. Comparative protein expression profiles of *Arthrospira (Spirulina) platensis* (ASP) under temperature stress for 7 days were also analyzed using 2DE technology [26]. The results showed that proteins involved in post-translational modification, energy metabolism, translation, and carbohydrate metabolism play predictable roles in ASP resistance to temperature stress. Nevertheless, most of the proteomics researches have been limited to the use of 2D gel electrophoresis. Because most of the hydrophobic and low-abundant proteins cannot be detected with 2D gel technologies, its application to the comprehensive analysis of proteomics changes is limited [27–29]. Isobaric tags for relative and absolute quantification (iTRAQ) is currently considered as one of the most robust techniques that allows the quantification of proteins; the technique utilizes peptide labeling and enables the identification and accurate quantification of proteins from multiple samples within wide dynamic ranges of protein abundance including hydrophobic and low-abundance proteins. Moreover, iTRAQ can be utilized for quantitative and qualitative research studies on proteins involved in post-translational modification such as phosphorylated proteins and glycosylated proteins [30–33].

In the present study, the physiological and proteomic responses of *Spirulina* to LT stress were analyzed. iTRAQ was adopted for the analysis of the protein profiles of *Spirulina* under LT stress via transcriptome sequencing. The present study provides a comprehensive analysis of *Spirulina* under LT stress, which in turn allows a better understanding of how various proteins and their associated metabolism pathways are utilized in response to LT stress.

## Materials and Methods

### Culture conditions and sample preparations

*Spirulina* was supplied by the Institute of *Spirulina* of the Beijing Forestry University (Beijing, China) and was cultured in Zarrouk medium at 30°C and 5 klx illumination intensity. At the logarithmic growth phase, cell samples were transferred into Zarrouk’s medium at different temperatures (15°C for LT and 30°C for control), and the initial inoculated concentration at a wavelength of 560 nm was 0.3. The cultures were conducted at each temperature for 24 h, and each treatment consisted of three independent biological replicates. Then, the filaments were collected and washed three times. The samples were rapidly immersed in liquid nitrogen for 15 min.

### Physiological analysis

Dry weight determination was used to evaluate cell growth. The filaments of the control and treatment group were collected and washed three times with distilled water, and then the cells were dried by using a vacuum freeze-dryer.

The contents of chlorophyll a and carotenoid (μg/mL) were determined using the method described by Harris [34]. Chlorophyll (Chl) was extracted from dry powder using 90% acetone and an ultrasonic cell disrupter. The extract was then centrifuged at 10,000 rpm at 4°C for 15 min and the supernatant was collected. The absorbance of the extract was measured at wavelengths of 663 nm, 646 nm and 470 nm using a UV-vis spectrophotometer.

The soluble sugar content was determined by using the anthrone method with slight modifications [35]. The dry powder (0.05 g) was placed in a test tube containing 5 mL of distilled water, and then heated in a boiling water bath for 30 min. The mixture was then centrifuged and the supernatant was collected and transferred into a fresh tube, and the pellet was extracted twice. The supernatant was used for the determination of soluble sugar content. The assay depends on the rate change at a wavelength of 630 nm using a UV-vis spectrophotometer. The reaction mixture contained 0.5 mL of the sugar extract, 1.5 mL of distilled water, 0.5 mL of anthrone ethyl acetate (1 g anthrone in 50 mL ethyl acetate), and 5 mL of concentrated sulfuric acid.

Free proline concentrations were determined using the ninhydrin reaction method as described by Tang et al. with some modifications [36]. The dry powder (0.05 g) was mixed in 5 mL of 3% sulfosalicylic acid in a tube and then heated in a boiling water bath for 15 min. After centrifuging at 4,500 rpm for 10 min, the supernatant was collected and mixed with an equal volume of acetic acid and acid ninhydrin, and then heated in a boiling water bath for 30 min. Four milliliters of toluene were added to the cooled solution, and color development of the extract was measured at a wavelength of 520 nm using a UV-vis spectrophotometer.

### Protein extraction

The samples were further ground in liquid nitrogen using a mortar. Next, five times the volume of trichloroacetic acid (TCA)/acetone (1:9) was added to the fine powder, subjected to vortex blending, incubated at -20°C for 4 h, and then centrifuged at 6,000*g* at 4°C for 40 min to collect the pellets. The pellets were resuspended in pre-cooled acetone, washed thrice, and then dried in a fume hood. Approximately 20–30 mg dry powder was then mixed with 30 times the volume of (m/v) the SDT lysates, resuspended by vortexing, and heated in a boiling water bath for 5 min. Ultrasonication (80 W, working: 10 s, interval: 15 s, looping: 10 times) was then performed, following by incubating in a boiling water bath for 15 min. The samples were centrifuged at 14,000*g* for 40 min, and the supernatant was filtered through a 0.22-μm filter membrane, and the supernatant was saved and stored at −80°C until analysis. The BCA method was used to qualify protein concentrations[37].

### Protein digestion and iTRAQ labeling

Protein extraction and digestion was performed using the FASP method as described by Wisniewski [38].The resulting peptide mixture was then labeled with the 8-plex iTRAQ reagent, according to the manufacturer’s instructions (AB Sciex Inc., Foster City, CA, USA). Briefly, 200 μg of proteins from each sample were added into 30 μL of an STD buffer (4% SDS, 100 mM DTT, 150 mM Tris-HCl pH 8.0). The detergent, DTT, and other small molecular substances were removed with UA buffer (8 M Urea, 150 mM Tris-HCl pH 8.0) by repeated ultrafiltration (micron units, 30 kD). Subsequently, 100 μL of 0.05 M iodoacetamide in UA buffer was used to block reduced cysteine residues, and then the samples were incubated for 20 min in darkness. The filters were washed with 100 μL UA buffer thrice, then with 100 μL DS buffer (50 mM triethylammonium-bicarbonate at pH 8.5) twice. The samples were digested with 2 μg trypsin (Promega) in 40 μL DS buffer and incubated overnight at 37°C, and the resulting peptides were collected by centrifugation. Finally, peptide content was measured based on UV light spectral density at a wavelength of 280 nm.

For labeling, each iTRAQ reagent was dissolved in 70 μL of ethanol and added to the respective peptide mixture. Each treatment consisted of two (Control) or three (LT) biological replicates. The samples were labeled as (Sample 1–1)-114 and (Sample 1–2)-115 for control, (Sample 2–1)-116, (Sample 2–2)-117 and (Sample 2–3)-118 for LT. All labeled samples were multiplexed and vacuum-dried.

### Peptide fractionation with strong cation exchange (SCX) chromatography

After labeling, the samples were fractionated using the SCX chromatography on an AKTA Purifier system (GE Healthcare). The dried sample was reconstituted and acidified with 2 mL of buffer A (10 mM KH_2_PO_4_ in 25% of ACN, pH 2.7) and loaded onto a PolySULFOETHYL 4.6 × 100 mm column (5 μm, 200 Å, PolyLC Inc., MD, USA). The peptides were eluted at flow velocity of 1 mL/min with a gradient of 0%–10% buffer B (500 mM KCl, 10 mM KH_2_PO_4_ in 25% of ACN, pH 2.7) for 2 min, 10–20% buffer B for 25 min, 20%–45% buffer B for 5 min, and 50%–100% buffer B for 5 min. The elution process was monitored by absorbance at a wavelength of 214 nm and fractions were collected every 1 min. The collected fractions desalted on C18 Cartridges (Empore^™^ SpirulinaE Cartridges C18 (standard density), bed I.D.: 7 mm, volume: 3 mL, Sigma). Each fraction was concentrated by vacuum centrifugation, followed by reconstitution in 40 μL of 0.1% (v/v) trifluoroacetic acid (TFA). The samples were prepared for the subsequent LC-MS/MS analysis.

### Liquid chromatography (LC)-electrospray ionization (ESI) tandem MS (MS/MS) analysis by Q exactive

The samples were analyzed on a Q Exactive mass spectrometer that was coupled to Easy nLC apparatus (Thermo Fisher Scientific). The peptide mixture was added into a C18-RP column (Thermo Scientific Easy Column, 10-cm long, 75-μm inner diameter, 3 μm resin) in buffer A (0.1% formic acid in water) and then separated with buffer B (80% acetonitrile and 0.1% formic acid) by IntelliFlow technology over 140 min. MS data was collected using a data-dependent top 10 method dynamically choosing the most abundant precursor ions from the survey scan (300–1,800 m/z) for HCD fragmentation. Determination of the target value was based on predictive Automatic Gain Control (pAGC).

### Sequence database search and data analysis

MS/MS data were searched using MASCOT engine (Matrix Science, London, UK; ver. 2.2) embedded into Proteome Discoverer 1.3 (Thermo Electron, San Jose, CA, USA) against the *Spirulina* database (47,062 sequences, depending on our whole transcriptome sequencing of *S*. *platensis*). For protein identification, the parameters were set as follows: Peptide mass tolerance at 20 ppm, MS/MS tolerance at 0.1 Da, trypsin enzyme with number of missed cleavage at 2, fixed modification: iTRAQ 8plex(K), iTRAQ 8plex(N-term), variable modification: oxidation(M). All data were based on at least one unique peptide with 99% confidence for protein identification and FDR ≤0.01.

### Bioinformatics analysis

The GO function entries for all alignment protein sequences were extracted using the mapping function of BLAST2GO (Version 3.0). Gene Ontology (GO) annotation was used to analyze the identified proteins, then the identified proteins were categorized by biological process, molecular function, and cellular component. The differentially expressed proteins were further analyzed by using the Kyoto Encyclopedia of Genes and Genomes (KEGG) database (http://www.genome.jp/kegg/kaas/).

Cluster 3.0 software was used to investigate the hierarchical clustering of the identified differentially expressed proteins. Java TreeView was used for data visualization. Columns were mean centered, and Euclidean distance and average linkage were used for data aggregation.

## Results

### Physiological response to LT stress

**Fig 1:**
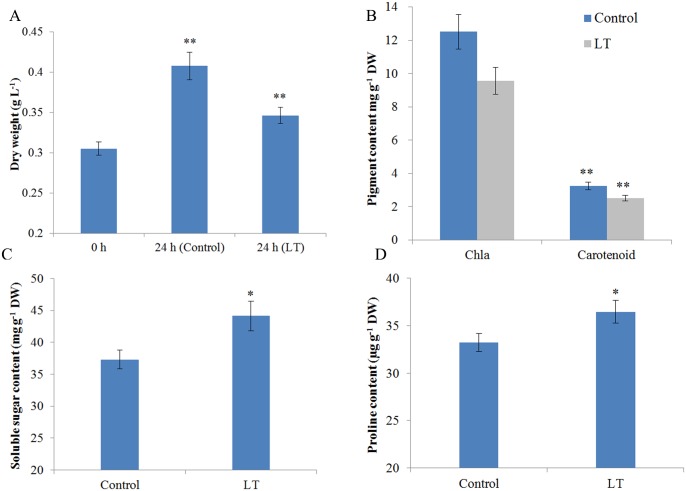
Physiological parameters of *Spirulina* under LT stress. (A) The dry weight of cells. (B) The contents of chlorophyll a and carotenoid. (C) Soluble sugar content. (D) Proline content. The values represent the mean ± SD of three biological replicates. The asterisks indicate the significant differences that were calculated using the t-test (*P < 0.05; **P < 0.01).

To study the physiological changes of *Spirulina* under LT, the cell culture from the control temperature (30°C) was exposed to LT (15°C) for 24 h. The results showed that the increment of cell dry weight decreased by 20.11% compared to that in the control group after 24 h of culture, thereby indicating poor growth under LT stress (Fig 1A). In addition, *Spirulina* cultures were less green in appearance compared to the control group, which was attributable to a decrease in chlorophyll a content. Chlorophyll a content was reduced by 23.61% compared to that in the control, and a similar decrease in carotenoid content was observed with a 22.53% reduction (Fig 1B). These results indicate that a significant loss of photosynthetic pigments. Accumulation soluble sugars and amino acids is a typical response of organisms to LT stress. We further determined the osmolyte levels under LT stress (Fig 1C and 1D). The soluble sugar and proline levels increased by 18.35% and 9.65% under stress, respectively.

### Quantitative proteomics analysis with iTRAQ

Temperature is a major abiotic stress affecting the survival and growth of cyanobacteria, including *Spirulina*. Over time, cyanobacteria have evolved a survival tactic to adapt LT by up-regulating and down-regulating specific target proteins. Therefore, these differentially expressed proteins play significant roles when responding to LT.

**Fig 2:**
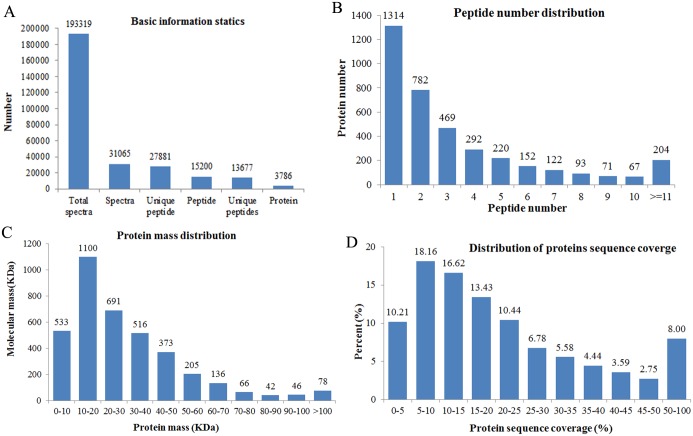
Identification and quantitative evaluation of identified proteins. (A) Spectra, peptides, and proteins identified using Mascot. (B) Number of peptides matching the proteins identified by using MASCOT. (C) Distribution of identified proteins according to different molecular masses. (D) Peptide coverage of the identified proteins.

The present study conducted an LT-induced proteomic experiment by iTRAQ labeling using *Spirulina*. The Mascot software was used to analyze the data, and a total of 193,319 spectra were generated from samples, with 31,065 unique spectra matching 15,200 peptides, 13,677 unique peptides, and 3,786 proteins (Fig 2A). The distribution of peptide number is shown in Fig 2B, with more than 60% of the proteins having at least two peptides. The protein mass distribution is shown in Fig 2C, which shows that approximately 86% of the MW range of the proteins were larger than 10 kDa, thereby indicating good coverage. Moreover, most of the identified proteins have good peptide coverage, and the sequence coverage of proteins more than 10% and 20% was 71% and 41%, respectively, thereby indicating high confidence (Fig 2D).

### Functional analysis of differentially expressed proteins

**Fig 3:**
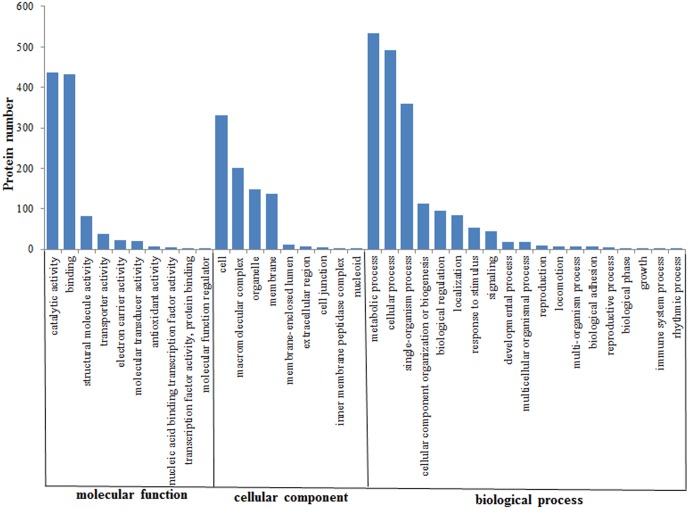
GO classification of differentially expressed proteins in *Spirulina* under LT stress.

**Fig 4:**
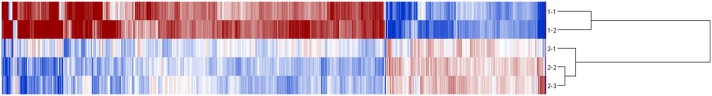
Hierarchical clustering of differentially expressed proteins. 1–1 and 1–2 represent two biological replicates of the control group. 2–1, 2–2, and 2–3 represent three biological replicates of the treatment group. The columns represent individual proteins, and rows represent treatment groups. Red and blue indicate up-regulated and down-regulated levels, respectively, and white indicates no significant changes in the expression levels of proteins in response to stress.

In the present study, proteins with relative abundances of > 1.2 and a P value < 0.05 were regarded as up-regulated, whereas those with a relative abundance < 0.833 and P value < 0.05 were down-regulated. A total of 1,062 proteins showed differential expression under LT stress and 313 proteins were up-regulated, whereas 749 proteins were down-regulated (S1 Table). More than 70% of the total number of differentially expressed proteins was down-regulated in the present study, and our physiological data also indicate that LT stress significantly reduces the growth rate of *Spirulina*. These findings indicate that LT has a significant effect on the protein expression profile of *Spirulina*. The GO database was used in the functional analysis of all differentially expressed proteins. GO analysis annotated 754 differential expressed proteins and further categorized these into three groups: biological process, cellular component, and molecular function (Fig 3). The results indicated that these proteins were involved in almost every aspect of *Spirulina* metabolism. The most predominant molecular function was catalytic activity and binding; other major functional categories were structural molecule activity and transporter activity. The most predominant cellular components were located in the cell, membrane, organelle, and macromolecular complex. The most prevalent biological processes categories were cellular process, metabolic process, single-organism process, and cellular component organization or biogenesis. To analyze the metabolism pathways that were involved in eliciting a response to LT stress, 324 differentially expressed proteins were further analyzed by using the KEGG database. The results showed that most of the proteins were enriched in the ribosome, biosynthesis of amino acids, carbon fixation in photosynthetic organisms, glycolysis/gluconeogenesis, and photosynthesis (Table 1). Cluster analysis of the differentially expressed proteins was performed using the protein abundance data using the cluster software. The results showed high reproducibility in protein relative abundance in the LT and control groups (Fig 4).

**Table 1:** KEGG pathway of most differentially expressed proteins.

Pathway	Number of proteins	Number of Up/Down regulated proteins	Pathway ID
Carbon metabolism	58	9/49	ko01200
Ribosome	54	11/43	ko03010
Biosynthesis of amino acids	46	11/35	ko01230
Glycolysis / Gluconeogenesis	27	8/19	ko00010
Purine metabolism	25	7/18	ko00230
Carbon fixation in photosynthetic organisms	22	6/16	ko00710
Pentose phosphate pathway	19	5/14	ko00030
Citrate cycle (TCA cycle)	19	2/17	ko00020
Glyoxylate and dicarboxylate metabolism	19	3/16	ko00630
Pyruvate metabolism	18	7/11	ko00620
2-Oxocarboxylic acid metabolism	17	2/15	ko01210
Photosynthesis	18	2/16	ko00195
Oxidative phosphorylation	17	4/13	ko00190
RNA degradation	16	6/10	ko03018
Starch and sucrose metabolism	12	4/8	ko00500
Porphyrin and chlorophyll metabolism	10	2/8	ko00860

## Discussion

Organisms have evolved a set of complex metabolism mechanisms to cope with LT stress. We performed a physiological and proteomics study of the response of *Spirulina* to LT stress to provide novel insights into the mechanism underlying LT tolerance in this species. The findings of the present study indicated that most of the identified differentially expressed proteins in the KEGG pathway were enriched in carbohydrate metabolism, photosynthesis, amino acid biosynthesis, and translation, which might be helpful in building a new metabolism balance in *Spirulina* under LT stress. The physiological results were consistent with the findings of our proteomics analysis. The significance of the putative role of the identified proteins and related metabolism pathway in LT stress will be discussed below.

### Proteins related to photosynthesis

Photosynthesis is highly susceptible to LT stress. In the present study, photosynthesis-related proteins were reduced under LT stress, which was accompanied by a significant loss of photosynthetic pigments (chlorophyll a and carotenoids). Photosystems I and II, cytochrome b6f complex and ferredoxin are the primary components involved in photosynthetic electron transfer, and 18 differentially expressed proteins involved in this light reaction process were identified (S2 Table). Photosystem I proteins (PsaC, PsaD, PsaE, and PsaF) and Photosystem II proteins (PsbC, PsbD, PsbO, PsbU, PsbV, and Psb28) were down-regulated under LT stress. In addition, components of cytochrome b6f complex and ferredoxin were all down-regulated following exposure to LT stress. The down-regulated proteins induced a decrease in the rates of electron transfer, which was subsequently followed by a reduction in photosynthetic rates. Thus, we concluded that the photosynthetic capacity of *Spirulina* was diminished due to a reduction in the expression of proteins are associated with the light reaction center. Furthermore, the observed decrease in the content of chlorophyll a and carotenoid were consistent with down-regulated of relevant biosynthesis proteins. Studies have shown that LT disrupts the balance between energy absorption through photosynthesis and energy utilization, and such conditions could produce a higher excitation pressure on PSII, which may be harmful to photosynthetic apparatus [39–41]. The loss of photosynthetic pigments affects the process of electron transfer, as well as reduces energy flux into the photosystems and prevents photodamage caused by absorbing excess light energy [20]. Our results show that the photosynthetic capacity of *Spirulina* was reduced under LT stress, and the decrease in the level of pigments may considered as a protective response to stress.

### Proteins related to carbon fixation in photosynthesis

**Fig 5:**
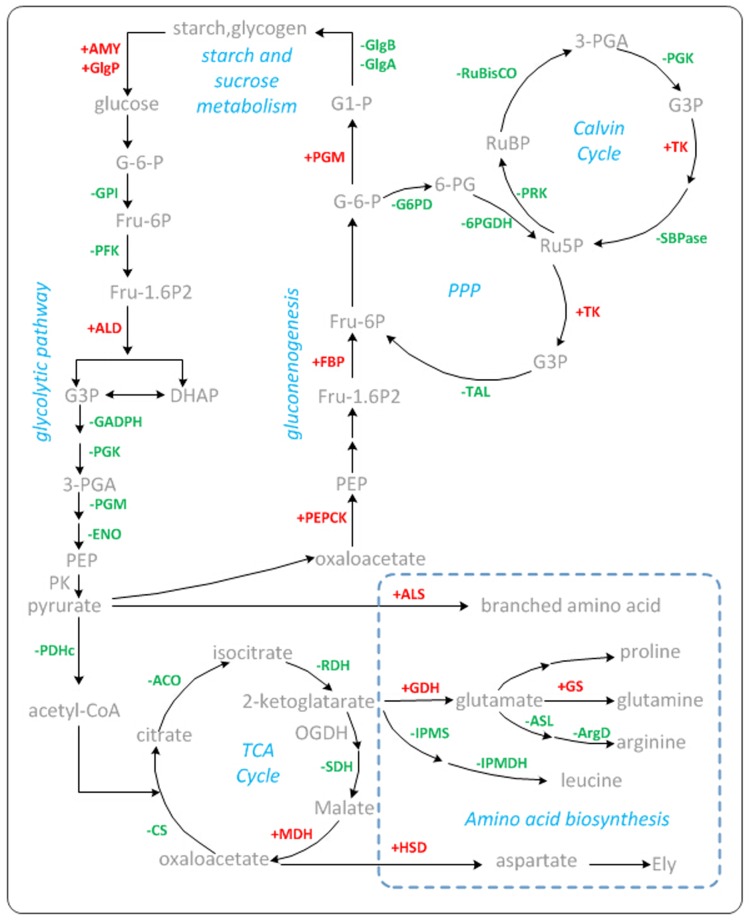
Carbon metabolism and amino acid biosynthesis are involved in the response of *Spirulina* to LT stress. Red and green numbers represent the up- and down-regulated proteins, respectively. The abbreviations for the proteins are as follows: AMY, alpha-amylase; GlgP, glycogen phosphorylase; GlgB, 1,4-alpha-glucan branching enzyme; GlgA, glycogen synthase; GPI, glucose-6-phosphate isomerase; PFK, 6-phosphofructokinase; ALD, fructose-bisphosphate aldolase; GADPH, glyceraldehyde-3-phosphate dehydrogenase; PGK, phosphoglycerate kinase; PGM, phosphoglycerate mutase; ENO, enolase; PK, pyruvate kinase; PDHc, pyruvate dehydrogenase complex; CS, citrate synthase; ACO, aconitate hydratase; IDH, isocitrate dehydrogenase; OGDH, oxoglutarate dehydrogenase complex; SDH, succinate dehydrogenase; G6PD, glucose-6-phosphate-1-dehydrogenase; 6PGDH, 6-phosphogluconate dehydrogenase; TK, transketolase; TAL, transaldolase; FBPase, fructose-1 6-bisphosphatase; PRK, phosphoribulokinase; ALS, acetolactate synthase; GDH, glutamate dehydrogenase; GS, glutamine synthetase; HSD, homoserine dehydrogenase; ASL, argininosuccinate lyase; ArgD, acetylornithine aminotransferase; IPMS, 2-isopropylmalate synthase; IPMDH, 3-isopropylmalate dehydrogenase.

Carbon fixation pathway produces the substrate for the synthesis of various carbohydrates and other metabolic products. A total of 22 proteins were identified to participate in this pathway, and the levels of several key enzymes involved in the Calvin cycle, including RuBisCO, sedoheptulose 1,7-bisphosphatase (SBPase), phosphoglycerate kinase (PGK), glyceraldehyde-3-phosphate dehydrogenase (GADPH), and phosphoribulokinase (PRK), were observed to decrease (Fig 5, S2 Table). RuBisCO (gene77537_2–0 and gene77440_1–0) consists of eight large subunits and eight small subunits and plays a critical role in photosynthetic carbon assimilation in the Calvin cycle [42]. A previous study suggested that RuBisCO is responsible in regulating carbon flux through the Calvin cycle when organisms subject to short-term environmental fluctuations [43]. This indicates that the degradation of the RuBisCO may be one of the major reasons behind the inhibition of carbon fixation upon exposure to LT. Sedoheptulose 1,7-bisphosphatase is also an important enzyme in controlling carbon assimilation in the Calvin cycle that affects the growth and development of organisms [44–46]. A small decrease in sedoheptulose 1,7-bisphosphatase activity could result in a significant reduction in carbon assimilation rate [47,48]. These findings support our speculation that down-regulated key enzymes in the Calvin cycle inhibited carbon assimilation during carbon fixation in photosynthetic organisms. Taken together, these findings indicate that the entire photosynthetic process of *Spirulina* was inhibited under LT stress.

### Proteins related to carbohydrate and energy metabolism

In our proteomic profile analysis, we identified 52 differentially expressed proteins involved in carbohydrate metabolism (Fig 5, S2 Table). Most of proteins involved in glycolysis, the TCA cycle, and pentose phosphate pathways were down-regulated, particularly several key proteins. For example, 6-phosphofructokinase is a key rate-limiting enzyme in the glycolytic pathway; it catalyzes the phosphorylation of fructose-6-phosphate to fructose-1,6-bisphosphate [49]. Citrate synthase catalyzes the conversion of oxaloacetate and acetyl-CoA into citrate and is the first rate-limiting enzyme in the TCA cycle [50]. 6-phosphogluconate dehydrogenase and glucose-6-phosphate-1-dehydrogenase are key enzymes in the pentose phosphate pathway. Most of the proteins related to oxidative phosphorylation were also down-regulated. Glycolysis, the TCA cycle, pentose phosphate pathway, and oxidative phosphorylation are major processes that are associated with respiration. The rate of respiration decreased when *Spirulina* under LT stress. Respiration is an important metabolic activity that is involved in the catabolism of carbohydrate, and is also a major route in providing energy for organisms. Under LT stress, the metabolism activity of *Spirulina* was reduced, and was accompanied by lower energy consumption, and a reduction in respiration may be helpful in energy maintenance.

Nevertheless, some important enzymes that participate in gluconeogenesis and starch and sucrose metabolism were up-regulated. Gluconeogenesis involves the conversion of non-sugar substances into sugar. Fructose-1,6-bisphosphatase and phosphoenolpyruvate carboxykinase (PEPCK), which are key enzymes in this metabolism pathway, were up-regulated. This indicated that gluconeogenesis is enhanced under LT stress in *Spirulina*. An increase in PEPCK occurs when plants are subjected to various abiotic stresses [51–53]. Studies have also shown that the increased abundance of the PEPCK protein or the overexpression of the PEPCK gene promotes soluble sugar accumulation in plants [54,55]. Therefore, PEPCK may play an important role in low temperature resistance in *Spirulina*. 12 enzymes involved in starch and sucrose metabolism were differentially expressed (Fig 5, S2 Table). Glycogen phosphorylase and α-amylase, which break down polysaccharides into monosaccharides, were up-regulated. These two proteins are also up-regulated in various species exposed to LT stress [56–58]. Moreover, some enzymes related to polysaccharides biosynthesis (maltooligosyl trehalose synthase, 1,4-alpha-glucan branching enzyme, glycogen synthase) underwent a decrease in levels. These findings indicate that LT tends to induce *Spirulina* to produce more soluble monosaccharides and oligosaccharides. This finding is also consistent with our results that the content of soluble sugar increased under LT stress. These soluble sugars can serve as osmolytes or substrates that are used for other biosynthetic process. The increase in soluble sugar content when plants are subjected to abiotic stress is a typical adaptive response [59], thereby those up-regulated proteins that are related to the increase of soluble sugar content may facilitate in improving LT tolerance in *Spirulina*. Several researches have demonstrated that LT acclimation increases the levels of soluble sugars, which is positively correlated with LT tolerance in organisms [60,61]. Hence, the content of soluble sugar may serve as a biochemical index to assess LT tolerance in *Spirulina*.

### Proteins involved in amino acid biosynthesis

Amino acids and other soluble nitrogenous compounds have been considered as the main product of inorganic nitrogen assimilation, and used for biosynthesis of protein and nucleic acids [62]. Previous studies have also indicated that amino acids could act as signaling molecules and osmolytes, regulating ion transport and facilitating detoxification when plants are subjected to stress conditions [63–65]. The present study identified a total of 46 proteins that were related to amino acid biosynthesis (Fig 5, S2 Table). Among these, the down-regulated enzymes mainly participate in the biosynthesis of leucine and arginine. Nevertheless, several identified key enzymes involved in amino acid biosynthesis were up-regulated, including glutamine synthetase (GS), glutamate dehydrogenase (GDH), acetolactate synthase (ALS), and homoserine dehydrogenase (HSD). GS and GDH are critical enzymes that can catalyze the conversion of ammonia into organic nitrogen. GDH enhances the production of glutamate, which is a precursor of proline biosynthesis, thereby leading to the production of more prolines. Our experimental results confirm the observed increase in proline content by 9.65% compared to that in the control. Proline is a well-known osmolyte that stabilizes biomacromolecules, scavenges ROS, and functions as a molecular chaperone to protect protein integrity and to enhance the activities of different enzymes under abiotic stress [66–68]. Studies have shown that the one of the most important roles of proline in LT stress may be that it combines with the hydrophobic domain of proteins, increases protein hydrophilicity, improves protein solubility, and further maintains the state of low temperature enzyme confirmation [68,69]. Therefore, the increase in proline content in *Spirulina* may be considered as protective mechanism against LT stress. GS has a high affinity for ammonia and plays an essential role for living cells to respond to a low concentration ammonia environment [70]. GS catalyzes the assimilation of ammonia with glutamate to yield glutamine (Gln). Gln serves as the substrate for the biosynthesis of organic nitrogenous compounds and other osmotic regulation substances [71]. These findings were further supported by the enhancement of GS activity and proline content in maize cultured cells under chilling stress [72]. ALS is a key enzyme that participates in the first step of branched chain amino acids biosynthesis (Ile, Leu, Val) [73]. HSD is rate-limiting enzyme in aspartate family amino acid biosynthesis, which controls the synthesis of methionine, and threonine[74]. We therefore can conclude that the increased abundance of these key enzymes is helpful in alleviating the inhibition of LT in the amino acid biosynthesis pathway, thereby facilitating the accumulation of more specific amino acids (e.g., glutamate and proline). The increase in the synthesis of amino acids on the one hand, as the osmolytes to maintain osmotic balance; on the other hand, they have the ability to maintain the dynamic balance of cell components’ damage and repair, to alleviate the injury degree of organisms, and to improve the LT tolerance in *Spirulina*.

### Proteins involved in translation

Our proteomic analysis identified 54 differentially expressed ribosome subunits, of which almost 80% (43/54) were down-regulated under LT stress (S2 Table). Ribosomes are sensitive to the environmental changes, and slight temperature changes lead to a rapid but temporary repression of the ribosome protein subunit (RPs) genes, consequently affecting ribosomal proteins [75]. In addition, some up-regulated subunits such as L18, L22, L24, and S5 that were initially exclusively associated with the synthesis of proteins may have additional or alternative roles, including acting as regulatory proteins or forming complexes with other cellular constituents [76,77]. Aminoacyl-tRNA synthetases and elongation factors also play significant roles in protein synthesis, and all of these were down-regulated under LT stress (S2 Table). The global decrease in the production of the protein synthetic machinery is a major contributing factor to slowing down or inhibiting the growth rate of *Spirulina* under LT. Our physiological study confirmed that the growth rate of *Spirulina* significantly decreased with LT treatment (15°C).

## Conclusions

In the present study, physiological and proteomic profiling of *Spirulina* under LT stress was performed. Proteomic analysis presents abundant information on individual proteins and relevant biological processes. An iTRAQ-based proteomic analysis technology was performed to analyze proteomics changes under LT stress in *Spirulina*. In the present study, we identified 1,062 differentially expressed proteins. GO and KEGG pathway analyses showed that most of differentially expressed proteins were involved in photosynthesis, carbohydrate metabolism, amino acid biosynthesis, translation as major response mechanisms to reconstruct cellular homeostasis and metabolic balance under LT stress. The up-regulated proteins involved in gluconeogenesis, starch and sucrose metabolism, and amino acid biosynthesis may have an active role in the response to stress. In contrast, the down-regulated expression of proteins related to photosynthesis, respiration, and translation may be required to reduce energy consumption for maintaining survival and growth of *Spirulina* under LT stress. In addition, our proteomics data were supported by the results of our physiological experiments. In conclusion, the results of our comprehensive proteomic analysis of *Spirulina* under LT stress improve our understanding of the proteins involved in the metabolic pathway response to stress. Further studies on target proteins and gene function analysis to elucidate the molecular mechanism underlying the response of *Spirulina* to LT stress are warranted.

## Supporting Information

S1 Table Differentially expressed proteins identified by iTRAQ. (XLS)

S2 Table The expression level of differentially expressed proteins in KEGG metabolism pathway. (XLS)
