# Correction: iTRAQ-Based Quantitative Proteomic Analysis of *Spirulina platensis* in Response to Low Temperature Stress

**DOI:** 10.1371/journal.pone.0196442

**Published:** 2018-04-23

**Authors:** Qingye Li, Rong Chang, Yijun Sun, Bosheng Li

The first two authors, Qingye Li and Rong Chang, should be noted as contributing equally to this work.
